# Anterior Cruciate Ligament Reconstruction Utilizing Double Adjustable-Loop Suspensory Fixation Devices Provides Good Clinical Outcomes in Patients under the Age of 40 Years at Two-Year Follow-Up

**DOI:** 10.3390/jcm13185436

**Published:** 2024-09-13

**Authors:** Theofylaktos Kyriakidis, Alexandros Tzaveas, Ioannes Melas, Kosmas Petras, Artemis-Maria Iosifidou, Michael Iosifidis

**Affiliations:** 1 Department of Orthopaedic Surgery and Traumatology, Erasme University Hospital, Université Libre de Bruxelles, Route de Lennik 808, 1070 Brussels, Belgium; the.kyriakidis@gmail.com; 2 2nd Orthopaedic Department, General Hospital “G. Gennimatas”, Aristotle University of Thessaloniki, 54635 Thessaloniki, Greece; 3 3rd Orthopaedic Department, Interbalkan Medical Center, 57001 Thessaloniki, Greece; tzaveas@alexandrostzaveas.gr (A.T.); im@imos.gr (I.M.); petraskosmas81@gmail.com (K.P.); 4 Orthobiology Surgery Center, 54623 Thessaloniki, Greece; 5 Medical School, Faculty of Health Sciences, Aristotle University of Thessaloniki, 54124 Thessaloniki, Greece; artemimi@auth.gr

**Keywords:** adjustable loop, anterior cruciate ligament reconstruction, suspensory devices, clinical outcomes, double adjustable-loop fixation

## Abstract

**Background:** Anterior cruciate ligament reconstruction (ACLR) using double adjustable fixation gained popularity in the last decade due to its minimally invasive technique. However, suspensory fixation devices could be related to recurrent instability, poor clinical outcomes, and patient dissatisfaction. The present study aims to evaluate the clinical outcomes following ACLR using double adjustable-loop suspensory fixation devices in the demanding population of young patients. **Methods:** Between 2019 and 2022, 95 patients with knee post-traumatic anterior cruciate ligament insufficiency were treated with primary ACLR using semitendinosus quadrupled graft and double adjustable-loop suspensory fixation devices and followed for at least two years. Concomitant lesions were also treated at the same surgical time. The knee examination form of the International Knee Documentation Committee (IKDC) was used to assess clinical evaluation, and the return to physical activities using the Tegner Activity Scale was recorded. Patient-reported objective measures (PROMs) were also evaluated, including the IKDC subjective and Lysholm scores. **Results:** Sixty-six males and twenty-nine females with a mean age of 23.8 (range 18–37) and a mean BMI of 24.9 (SD ± 2.42) kg/m^2^ were included in this study. All patients were evaluated clinically as normal or nearly normal at the final follow-up. PROMs also significantly improved postoperatively (*p* < 0.05) compared to the preoperative values. The Tegner Activity Scale increased from 2 to 7, the IKDC mean score improved from 43.9 (±8.9) to 93.3 (±12.3), and the modified Lysholm from 47.3 (±11.1) to 92.9 (±16.6). No complications or adverse events were recorded. **Conclusions:** Anterior cruciate ligament reconstruction utilizing double adjustable-loop suspensory fixation devices provides good clinical and functional outcomes in young patients at a two-year follow-up.

## 1. Introduction

Anterior cruciate ligament (ACL) rupture is one of the most common musculoskeletal injuries in cutting and pivoting sports, with an annual incidence rate of 68.6 per 100,000 person-years, especially in young people. It usually leads to symptomatic knee instability, and surgical treatment is generally utilized to perform anterior cruciate ligament reconstruction (ACLR) [1,2]. ACLR is widely used, aiming to restore the native knee joint anatomy, kinematics, and kinetics. However, some patients still report residual knee joint instability even after a successful ACLR [3,4]. Previous studies have reported that residual instability can lead to graft failure, osteochondral damage, and knee joint osteoarthritis [5], significantly affecting patients’ daily activity and quality of life [6,7].

Autologous grafts are the most common grafts during the ACLR procedure, and in the last twenty years, the hamstrings have gained popularity over the bone-patellar tendon-bone (BPTB). They present numerous advantages as they are easy to harvest, have less donor site morbidity, and do not generate anterior knee pain. Moreover, they have a force similar to the native ACL, easy graft passage, and fast acquisition. On the other hand, hamstrings are associated with an increased risk of infection compared to BPTB, unexpected size, and mainly decreased flexion and internal rotation strength [8].

Stable fixation and securing the graft without displacement in the tunnels are crucial for a successful surgical outcome and for avoiding residual laxity [9]. They permit early rehabilitation and functional and biological graft survivorship [10]. To date, various graft fixation techniques have been described in the recent literature. Traditionally, hamstring fixation was achieved with fixed-loop cortical devices (FLDs) in the femoral site and interference screws in the tibial socket with satisfactory clinical and functional results.

However, femoral tunnel widening was occasionally detected, probably due to graft motion, as a fixed-loop technique requiring additional drilling of the femoral tunnel to “allow” the button to come out of the lateral femoral cortex [11,12]. To overcome this technical limitation, adjustable-loop suspensory devices (ALDs) were used to minimize the “empty” space and fit the graft to the tunnel depth, which also had satisfactory outcomes in the recent literature [13].

Looking for less invasive surgical approaches with fewer donor-site morbidities and faster postoperative recovery, some authors proposed using an adjustable-loop device also for tibial fixation. This technique—which was named “all-inside ACLR”—preserves bone stock, avoids open tunnels, allows gracilis preservation, and permits adjustment of graft tension [14,15].

It also offers a shorter but larger diameter graft, which probably answers the graft-size considerations. Current evidence reports that graft size is essential to avoid failures. Indeed, biomechanical studies have demonstrated that the strength of a tendon graft is related to its diameter and that the thinner graft is correlated with a greater likelihood of graft rupture and joint instability [16]. Nevertheless, the exact graft diameter is unclear and also depends on other factors. Newer studies suggest that even enlargements of 0.5 mm up to a graft size of 10 mm benefit the patient. However, no evidence exists to recommend using grafts >10 mm. Other factors should be taken into consideration, mainly age. Hence, a bigger graft size should not be considered a unique ACL reconstruction goal [16]. In a systematic review and meta-analysis, Conte et al. found that there is a 6.8 times greater relative risk of failure for grafts less than 8 mm in diameter. They concluded that in hamstring ACL reconstruction, to decrease the risk of failure, a graft of at least 8.5 mm should be used [17].

Recent research has demonstrated that ACL reconstruction with an all-inside quadrupled semitendinosus autograft construct is equivalent to the historically considered “gold standard” bone-patellar tendon-bone (BPTB) autograft based on KT-1000 stability testing in athletes 24 years or younger [18]. Additionally, gracilis preservation is critical for postoperative rehabilitation, especially for patients requiring knee joint movement. Moreover, the all-inside technique requires a minimally invasive procedure in the tibia that reduces the incidence of possible complications such as tibial plateau fractures [19].

However, no consensus exists in the recent literature regarding the efficacy of adjustable loops. Some studies presented good clinical outcomes and MRI images demonstrating the graft’s healing and integration. Putnis et al. [20] aimed to evaluate the clinical outcome and MRI appearance after hamstring autograft ACLR using both femoral and tibial adjustable cortical suspension fixation. They found significant improvements in all clinical scores. The MRI analysis showed 71% of cases with fully integrated grafts in the tibia and 24% in the femur, with the remainder all showing higher than 50% integration. They concluded that ACLR using femoral and tibial adjustable suspensory fixation has good clinical results, with significant PROM improvement at one and two years and a low graft rupture rate. Additionally, grafts heal in most cases with comparatively low tunnel widening. Elmholt et al. [21] performed a systematic review and a meta-analysis, including 15 studies and 2686 patients. The primary outcome was to assess the risk of revision ACLR between ALDs and FLDs. Secondary outcomes were knee stability and PROMs. They reported no difference in revision rates between ALDs and FLDs in the included studies. Furthermore, the meta-analysis showed no differences regarding knee laxity and PROMs. They concluded that both types of loop devices are safe to use in ACLR.

On the other hand, several biomechanical studies demonstrated that adjustable-loop devices may lead to graft laxity as the loop is lengthened under cyclic loading [22,23]. Consequently, this could provoke recurrent instability and, perhaps, poor clinical outcomes or patient dissatisfaction. Jin et al. [24], in their biomechanical study, compared three cortical suspensory devices, one with a fixed loop and two with an adjustable loop, and they concluded that the fixed-loop devices showed significantly better mechanical properties in failure load and displacement than the adjustable-loop devices. Eguchi et al. [25] aimed to evaluate the mechanical strength of two cortical suspension devices, an adjustable and a fixed-length loop. The devices were tested under cyclic and pull-to-failure loading conditions. The results of their study indicated that the FLD provides greater mechanical strength than the ALD.

However, the clinical outcomes of an ACLR with double adjustable-loop suspensory fixation devices have not yet been sufficiently investigated. The purpose of the present study was to evaluate the clinical outcomes at a two-year follow-up. The hypothesis was that all patients would show improved functional outcomes.

## 2. Materials and Methods

### 2.1. Population

The present research is a retrospective study with an analysis of prospectively collected data. Between 2019 and 2022, 95 patients with knee post-traumatic anterior cruciate ligament rupture were treated with primary ACLR using semitendinosus quadrupled graft and double adjustable-loop suspensory fixation devices and followed for at least two years. Concomitant lesions were also treated at the same surgical time. Patients’ demographic characteristics are shown in Table 1.

The inclusion criteria were as follows: 1. male and female adults under 45 years; 2. isolated ACL rupture or associated with meniscal injury; 3. a minimum of two years’ follow-up; 4. semitendinosus quadrupled graft fixed with femoral and tibial suspensory buttons.

On the other hand, the exclusion criteria consisted of the following: 1. ACL revision procedure; 2. combined lateral extra-articular tenodesis (LET); 3. any previous knee operation; 4. bilateral ACLR.

### 2.2. Surgical Technique

**Figure 1:**
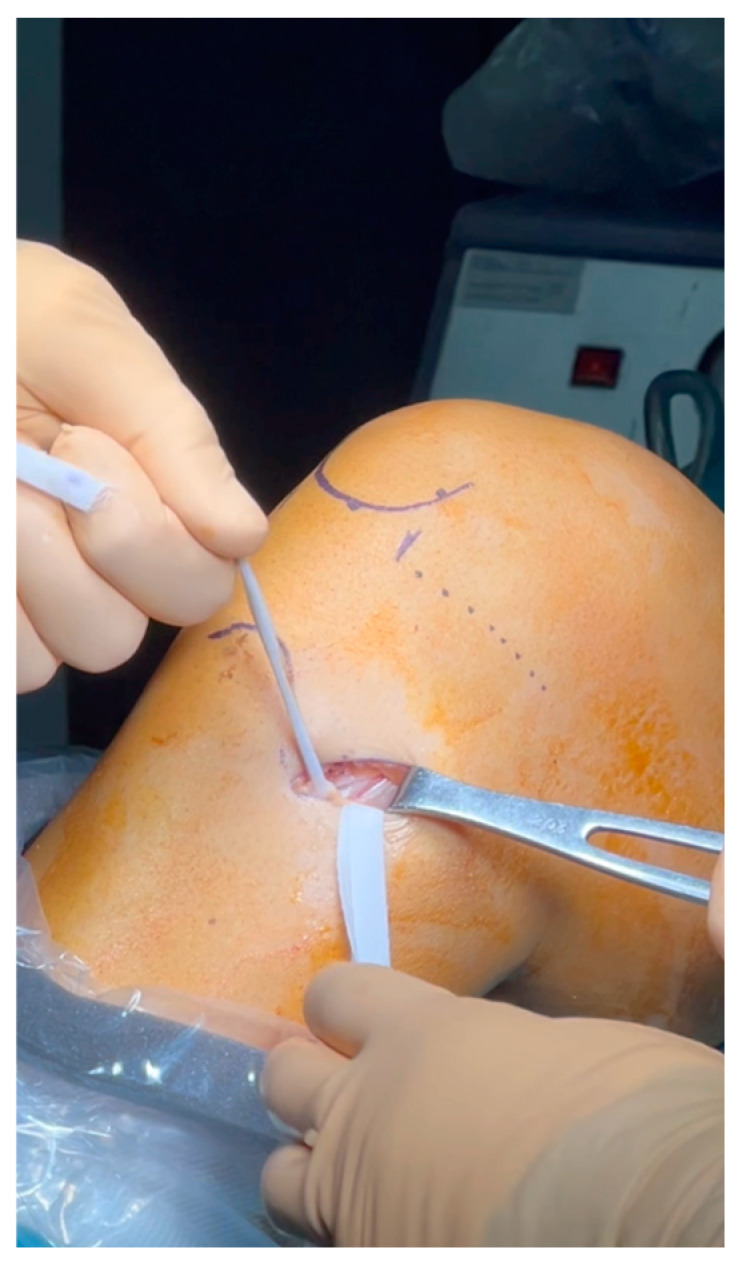
Minimally invasive harvesting technique.

**Figure 2:**
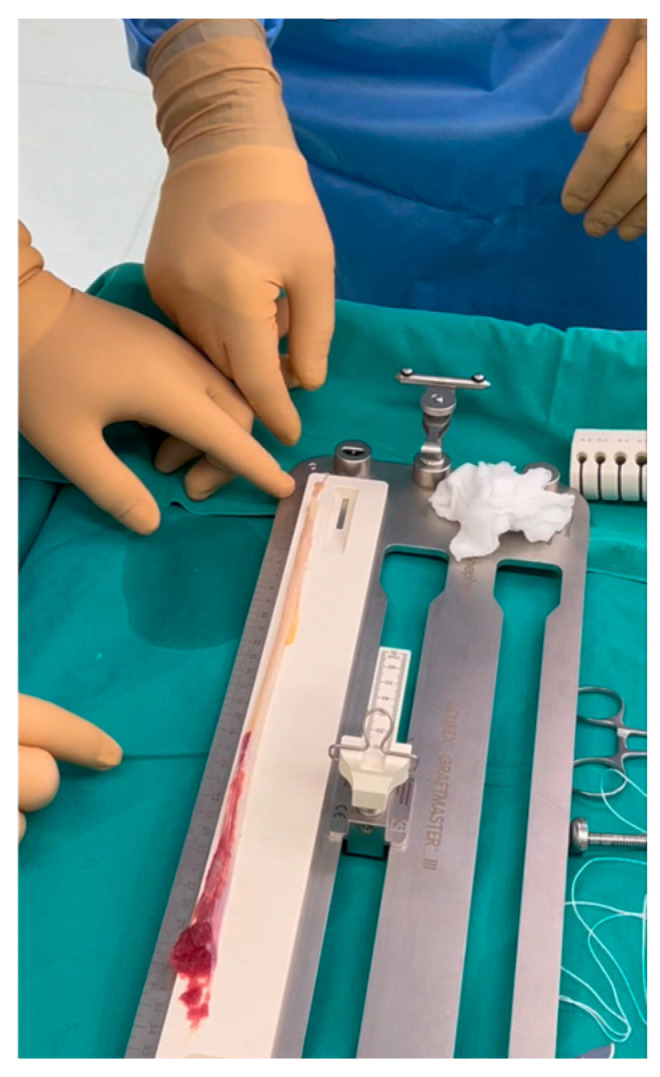
Harvested semitendinosus graft (total length should be at least 26 cm).

**Figure 3:**
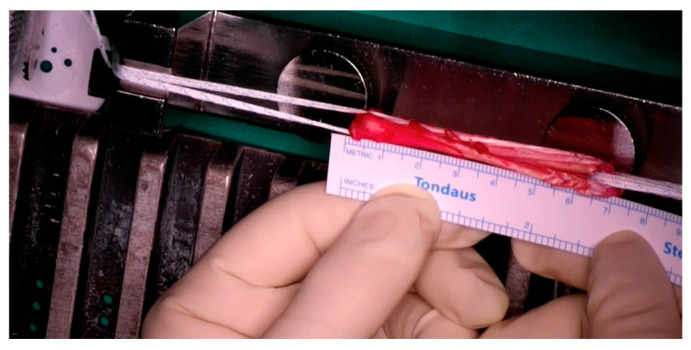
Four-strand graft (final construct of at least 6.5 cm).

Two specialized knee surgeons performed all operations. The procedure was conducted with the patient in a supine position under general anesthesia and a tourniquet application. An initial arthroscopic evaluation was conducted to estimate ACL tears and other intra-articular lesions. After the ACL deficiency verification, only the semitendinosus tendon was harvested, using a minimally invasive hamstring harvest technique with the required instruments (Figure 1). The overall graft length measured at least 26 cm (Figure 2) and yielded a 4-strand construct of at least 6.5 cm (Figure 3), providing a minimum of 2 cm of graft to be placed in each femoral and tibial socket. Two different surgical techniques were used for the ACLR after that point during the procedure.

**Figure 4:**
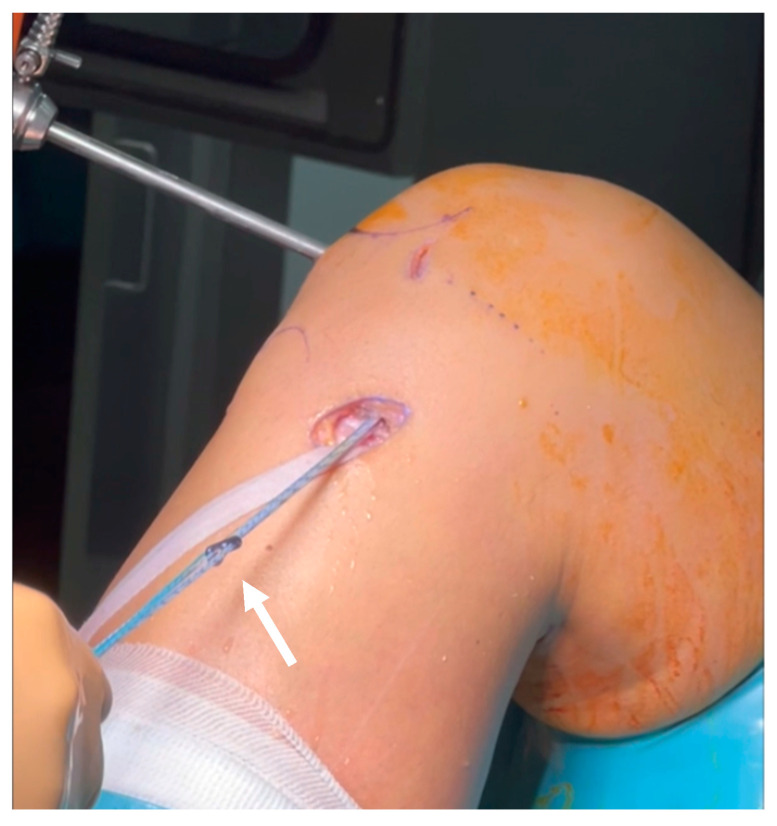
Arrow: Ultrabutton^®^ (Smith & Nephew).

The all-inside technique started with standard femoral tunnel formation based on anatomical landmarks of ACL insertion in the medial wall of the lateral femoral condyle. It was always performed from the medial portal. The anatomical femoral ACL insertion was marked, and a pin guide (2.5 mm) was inserted with the knee in full flexion. A conventional cannulated drilling device was used over the pin for preforming the full tunnel for button advancement through the femur (from the joint out of the cortex, 4.5 mm in width) and one wider (according to graft’s width) for preparing the “blinded” femoral tunnel (depth was up to 20 mm). For the tibial tunnel, the insertion site was also marked by the ACLR aiming guide for a tibial tunnel. Through this guide the specific retrograde drilling device for the all-inside technique was inserted in the anatomical position of the ACL tibial insertion. After that, this device’s edge was “advanced” to become a drill of the appropriate width (according to graft’s width), ready to perform the retro-drilling (from the joint) up to the premeasured depth (for receiving the graft). A suture loop was inserted from the medial portal to the femoral tunnel (for pulling the graft in the femur “as usual”), and another suture loop was also inserted through the tibial tunnel—from the cortex in the joint—and pulled out from the medial portal (for pulling the graft in the tibia). Then, one edge of the graft was pulled through the anteromedial portal to the femoral tunnel, and femoral fixation was completed with the adjustable-loop advancement. The other edge of the graft was also pulled through the medial portal, putting the graft in the tibial tunnel, and the tibia’s adjustable button appeared out of the cortex “ready” for fixation. Finally, the graft was fixed in the tibia by advancing the second adjustable-loop button on the tibia cortex. For securing the graft in both cortexes the following adjustable fixation devices were used: TightRope^®^ (Arthrex, Munich, Germany) and Ultrabutton^®^ (Smith & Nephew, Andover, MA, USA) (Figure 4).

**Figure 5:**
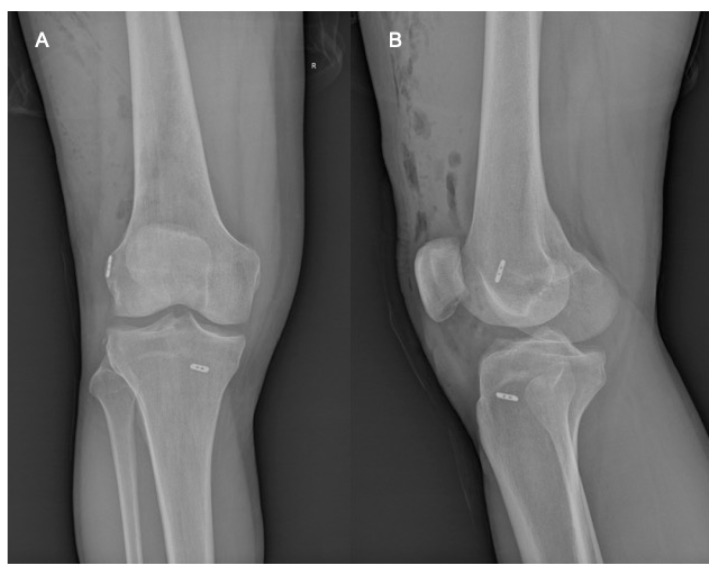
Post-operative X-rays, Aanteroposterior (**A**) and lateral (**B**).

The technique proposed by Silva [26] was also started with femoral tunnel preparation in exactly the same way that was described above. The tibial insertion site was also marked by the ACLR aiming guide for a tibial tunnel, and a pin guide (2.5 mm) was inserted. After weakening the cortex at the distal end of the tibial tunnel, a special bone dowel was used, and the distal part of the tunnel was removed en bloc (as a cylindrical bone plug of a length up to the “end” of the necessary tibial tunnel length for the graft). Finally, with a conventional cannulated drilling device (according to graft’s width), the rest of the tibial tunnel was reamed through the subchondral bone. The suture loop that had been inserted from the medial portal to the femoral tunnel, was then pulled from the joint out of the tibial tunnel. Finally, the graft was pulled through the tibial tunnel to the femoral tunnel (“as usual”). The femoral fixation was completed through the adjustable-loop advancement (ToggleLoc^®^-Zimmer-Biomet, Warsaw, IN, USA), which was followed by the advancement completion of the second adjustable-loop button for the tibia (the specific one for the tibia, which is wider than usual, (ToggleLocXL^®^-Zimmer-Biomet, Warsaw, IN, USA). The bone plug was finally compacted into the tibial tunnel exit after graft passage. Coexisting meniscal tears were treated simultaneously with partial meniscectomy using a shaver and punch or meniscal repair using all-inside and inside-out devices/sutures. Radiological postoperative evaluation was performed routinely with anteroposterior and lateral standing position views (Figure 5).

### 2.3. Evaluation of Outcomes

Data collection for the follow-up examination period was conducted prospectively, including the International Knee Documentation Committee (IKDC) objective score, which assessed clinical evaluation, and the return to physical activities score using the Tegner Activity Scale. Analytically, the IKDC examination form contains items that fall into one of the following seven measurement domains: effusion, passive motion deficit ligament examination, compartment findings, harvest site pathology, X-ray findings, and functional test. There are four evaluation grades: A: normal, B: nearly normal, C: abnormal, and D: severely abnormal. The lowest grade within a group determines the group grade, and the worst group grade determines the final evaluation of the patient. The Tegner Activity Scale graded patients’ activity based on work and sports activities from 0 to 10. Zero represents disability because of knee problems, and ten competitive sports at the national or international level. Patient-reported objective measures (PROMs), containing the IKDC subjective questionnaire and the modified Lysholm score, were also evaluated. The IKDC subjective form evaluates knee assessment based on questions about symptoms, sports activities, and function. The score ranges from 0 points, corresponding to the lowest level of function or the highest level of symptoms, to 100 points, equivalent to the highest level of function and the lowest level of symptoms. The modified Lysholm score evaluates knee function; the maximum score is 100 points: 91 to 100 points are considered excellent; 84 to 90, good; 65 to 83, fair; and 64 or less, unsatisfactory. Minor and major complications were equally recorded. The major complication was defined as the necessity for revision surgery due to recurrent instability.

### 2.4. Statistical Analysis

An independent statistician conducted a statistical analysis using SPSS 26.0 (IBM Corp, Armonk, NY, USA). The mean and standard deviation (SD) for all values are provided. The Shapiro–Wilk test was used to check if the data were normally distributed. The paired *t*-test for normally distributed data and the Wilcoxon signed-rank test for non-normally distributed data were performed to analyze outcomes between preoperative and postoperative findings. *p*-values less than 0.05 were considered statistically significant.

## 3. Results

**Figure 6:**
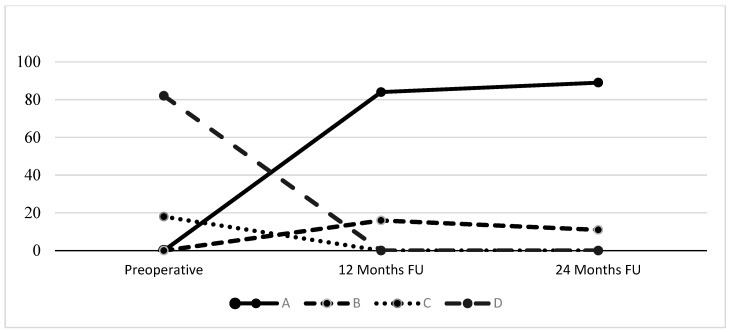
Evolution of the IKDC objective evaluation form. All values are presented as percentages.

The present study includes sixty-six males and twenty-nine females who met the eligibility criteria and had a mean age of 23.8 (range 18–37) and a mean BMI of 24.9 kg/m^2^. Most injuries involved the right knee, 64%, and 36% the left knee. In total, 38 cases presented concomitant meniscal injuries. Twenty-one were unrepairable tears and treated with partial meniscectomy. Ten and seven cases were longitudinal and bucket-handle tears, respectively, and had meniscal repair using all-inside devices and inside-out devices/sutures (3–6 sutures). The graft size was between 8.5 mm and 10 mm. All patients had sports-related injuries, mostly soccer-related, followed by skiing and basketball. The number of the three different suspensory loop devices used was split as follows: 44 TightRope^®^, 33 ToggleLoc^®^, and 18 Ultrabutton^®^. All patients presented statistically significant improvement over time in clinical and functional outcomes. More precisely, the clinical evaluation using the IKDC examination form revealed that the entire cohort was classified as normal in 89% and nearly normal in 11% of the cases (*p* < 0.05) at the two-year follow-up compared to the preoperative percentage of 82% severely abnormal and 18% abnormal. Tegner’s Activity Scale improved significantly, reaching level seven at the last evaluation, having started at level two at baseline (*p* < 0.05). The PROMs demonstrated equally increased values, with a mean IKDC subjective score rising from 43.9 (±8.9) to 93.3 (±12.3) and the modified Lysholm from 47.3 (±11.1) to 92.9 (±16.6). No complications or adverse effects related to treatment were recorded. The detailed results are presented in Figure 6 and Table 2.

## 4. Discussion

The most important finding of this study is that ACLR utilizing double adjustable-loop suspensory fixation devices is an efficient treatment option that provides anteroposterior knee stability and significantly improves clinical and functional outcomes. Furthermore, it is a safe procedure, with no complications or adverse effects related to the surgery.

The purpose was to evaluate the clinical and functional outcomes of patients who underwent all-inside ACL reconstruction. The study’s novelty and clinical relevance were due to the utilization of a technique that has not yielded sufficient results in the recent literature. The clinical assessment using the IKDC examination form presented a statistically significant improvement at the final two-year follow-up evaluation in the current study. Moreover, the IKDC, the modified Lysholm, and the Tegner Activity scores were significantly higher than the baseline. These results considered the clinical relevance of the study and demonstrated that double-adjustable devices for graft fixation report similar results to those observed in other surgical fixation techniques [27,28,29].

Several previous studies using the all-inside technique reported outcomes comparable to those of the present study. For instance, Schurz et al. [30] evaluated the clinical and functional results of 79 patients after anatomic ACL reconstruction using the all-inside technique with a minimum follow-up of 24 months. They concluded that the IKDC, Lysholm, VAS, and Tegner Activity scores significantly improved. Similarly, Colombet et al. [31] evaluated the clinical and functional outcomes of ACLR at a minimum of two years using adjustable suspensory fixation in both the femur and tibia in 97 cases. They concluded that the novel adjustable-length cortical suspensory fixation (CSF) device produced satisfactory anterior laxity and clinical outcomes, with a failure rate of 2.1%, which compares favorably with those reported for nonadjustable CSF devices. Kyriakopoulos et al. [32] compared the failure rates and clinical and functional outcomes of the all-inside ACLR with double suspensory fixation and quadrupled semitendinosus autograft with the anteromedial portal doubled semitendinosus–gracilis autograft with suspensory femoral and tibial interference screw fixation. They reported that the all-inside technique is equivalent in terms of outcomes to the traditional method and, given its less invasive nature and versatility in graft choices, it is a safe and effective technique for primary ACL reconstruction at three years’ follow-up.

Comparable to the results of the present study, in recently published research by Cai et al. [33], 78 young patients were followed up for at least two years. Functional recovery and pain relief were assessed using different PROMs, including IKDC, Lysholm, KOOS, and VAS. Instrumented laxity was assessed via side-to-side difference using an arthrometer. Their study also estimated graft maturity using the signal-to-noise quotient value based on magnetic resonance imaging. They reported that most patients with ACL rupture showed significantly better knee function, reduced knee laxity, satisfactory graft maturity, and significantly lower pain levels. Hence, using the all-inside technique, ACLR offers promising results. Other studies compared the MRI-based graft maturity characteristics of all-inside and standard single-bundle ACLR. Lin et al. [34] randomly and equally assigned fifty-four patients to an all-inside or standard reconstruction group. Their findings reported that both techniques exhibited poor maturity in the middle graft region and best in the distal region. Graft maturity with all-inside ACLR was inferior to that with standard ACLR in the early postoperative stage.

Adjustable suspensory devices at both the femur and tibia benefit from a short but larger diameter graft and many other advantages, such as gracilis preservation, less pain, and faster recovery. Nevertheless, some biomechanical studies [22,35] demonstrated that adjustable loops could generate recurrent instability and prompt graft failure. In the present study, all patients were classified clinically as normal or nearly normal, with no failures. These findings imply that double suspensory devices provide a stable graft fixation, as mentioned in previously published studies [31].

Various studies compared the all-inside technique to the complete tibial tunnel techniques in anterior cruciate ligament reconstruction. Kouloumentas et al. [36] compared in terms of clinical and functional outcomes the all-inside technique for ACLR using a short, quadrupled semitendinosus tendon autograft and suspensory cortical fixation on both the femoral and tibial side to the conventional method using a semitendinosus-gracilis autograft fixed with a suspensory device on the femoral side and an interference screw on the tibial side. They randomized and prospectively followed 90 patients into two equal groups of 45 for two years. The Lysholm, IKDC, KOOS, and KSS scores between the two groups were without statistically significant differences. However, patients of the all-inside group had significantly higher mean limb symmetry index (LSI) in terms of flexor peak torque, time-to-peak, and total work at 180°/s and significantly better mean LSI for isometric flexor/extensor ratio at 90°/s. They settled that all-inside ACL reconstruction with a quadrupled ST autograft and cortical button fixation on both ends is a viable alternative to the conventional technique. It preserves knee flexor strength, which is advantageous, especially when treating athletes with ACL injuries. Goyal et al. [37] aimed to determine whether the all-inside single-bundle ACL reconstruction technique differs from the complete tibial tunnel technique regarding graft dimensions, functional outcomes, and clinical outcomes. They performed a prospective comparative study that included 80 patients with isolated ACL tears and divided them into two groups of 40 patients each. The quadrupled semitendinosus tendon graft was significantly thicker than the doubled semitendinosus and gracilis tendons graft. The study results showed that the all-inside technique has the advantage of using a single tendon graft, resulting in lesser early postoperative pain with similar clinical and functional outcomes compared to the complete tibial tunnel technique.

A recent systematic review and meta-analysis of randomized controlled trials performed by Lv et al. [38] indicated that the all-inside ACLR was superior in functional outcomes and tibial tunnel widening. However, all-inside was not entirely superior to complete tibial tunnel ACLR in knee laxity measurement and graft re-rupture rate. Another systematic review performed by Fu et al. [39] concluded that all-inside ACLR with suspensory cortical button fixation was not clinically superior to the full tibial tunnel with interference screw fixation in functional outcomes, knee laxity measured with an arthrometer, or re-rupture rate. However, the advantage of using suspensory cortical button fixation was that a thicker graft could be used for reconstruction and that it brought about less tibia tunnel widening compared with bioabsorbable interference screw fixation. A recent comparative study by Pautasso et al. [40] showed that the all-inside technique showed good postoperative results at medium-term follow-up. Thus, it could be a valuable solution for ACL reconstruction, especially in young patients, due to its being less invasive. Similarly, the present study performed with minimally invasive approaches for the tibial tunnel using two different surgical techniques: the retrograde drilling used in the “classic” all-inside technique and the procedure described by Silva [26], in which the cortex at the distal end of the tibial tunnel is removed, followed by the harvesting of a bone dowel that is compacted into the tibial tunnel after graft passage.

Graft choice rationale is also critical to successful surgery. The existing literature proposes an individualized graft choice. Selection must consider different elements, including patient, physician, and graft factors. Nevertheless, each graft decision presents advantages and disadvantages [41,42]. Hamstring autografts are most often used, followed by BPTB and quadriceps tendon [43]. When minimally invasive ACLR techniques are used, only the semitendinosus is harvested in most cases. Clinical studies have demonstrated good functional outcomes after a quadruple semitendinosus graft with cortical fixations. The technique provides shorter but thicker grafts. A graft less than 8 mm in diameter may lead to poorer outcomes and an increased risk of graft rupture, especially in young athletes. It has been documented that a quadruple semitendinosus graft usually has an approximate diameter of 8–9.5 mm [37,44]. Moreover, the procedure spares the gracilis tendon, which preserves the medial-side muscle and could improve recovery of knee flexion strength and function and limit donor-side morbidity [45]. Recent investigations noted more suitable knee flexion strength with the AI ACLR reconstruction than the traditional ACL reconstruction [46].

Knee stability also depends on the appropriate graft position and both the femur and the tibia entry points. However, no consensus exists in the current literature between transtibial and tibial tunnel-independent reaming methods [47]. The authors’ preferred technique is to perform an anatomic tibial-independent femoral tunnel based on the ACL footprint insertion.

This study has several limitations. First, it does not include a comparative group treated with ACLR using an interference screw at the tibia, which is still the traditional fixation method. A future randomized study comparing these fixation techniques should be performed to verify the results of the present study. Second, the relatively short follow-up evaluation period could overlook problems that may need a longer time to appear. Third, two different ACLR surgical techniques for the tibial tunnel were performed without a comparative analysis of these procedures. Fourth, knee postoperative stability was evaluated manually, and thus it could be less precise than using an arthrometer, which gives objective and more accurate information.

## 5. Conclusions

Anterior cruciate ligament reconstruction is conducted regularly to cope with knee post-traumatic instability. Graft fixation is crucial to achieve adequate clinical outcomes and rapid rehabilitation. The present study demonstrated that the ACLR technique using double adjustable-loop suspensory fixation devices provides good clinical and functional outcomes in patients under the age of 40 years at the two-year follow-up. All patients showed significant improvement; therefore, this surgical technique should be among the treatment options.

## Figures and Tables

**Table 1:** Patient’s demographic characteristics.

Participants	95
Gender	Female, *n* = 29 Male, *n* = 66
Age	23.8 years (range 18–37)
BMI	24.9 ± 2.42 kg/m^2^
Side	Right, *n* = 61 Left, *n* = 34
Concomitant Lesions	Meniscal tear (*n* = 38) Treated by Meniscectomy, *n* = 21 Meniscal Repair, *n* = 17

**Table 2:** Evolution of the patient-reported outcomes measures.

	Preoperative	12 Months FU	24 Months FU
Tegner Activity Scale	2	6	7
IKDC Score	43.9 (±8.9)	92.9 (±11.7)	93.3 (±12.3)
Lysholm Score	47.3 (±11.1)	92.8 (±16.9)	92.9 (±16.6)

All values are presented as mean and standard deviation.

## Data Availability

The data supporting the conclusion of this article will be made available by the corresponding author on request.
